# “Availability of healthcare providers for rural veterans eligible for purchased care under the veterans choice act”

**DOI:** 10.1186/s12913-018-3108-8

**Published:** 2018-05-29

**Authors:** Michael E. Ohl, Margaret Carrell, Andrew Thurman, Mark Vander Weg, Teresa Hudson, Michelle Mengeling, Mary Vaughan-Sarrazin

**Affiliations:** 1 VA Office of Rural Health (ORH), Veterans Rural Health Resource Center- Iowa City, Iowa City VA Medical Center, Iowa City, IA USA; 2 Center for Comprehensive Access and Delivery Research and Evaluation (CADRE), Mailstop 152, Iowa City VAMC, 52246m, Iowa City, IA USA; 3 Department of Internal Medicine, University of Iowa Carver College of Medicine, Iowa City, IA USA; 4 Department of Geographical and Sustainability Sciences, University of Iowa, Iowa City, IA USA; 5 Center for Mental Healthcare and Outcomes Research, Central Arkansas VA, Little Rock, AR USA

**Keywords:** Access, Veterans, Rural health

## Abstract

**Background:**

Military Veterans in the United States are more likely than the general population to live in rural areas, and often have limited geographic access to Veterans Health Administration (VHA) facilities. In an effort to improve access for Veterans living far from VHA facilities, the recently-enacted Veterans Choice Act directed VHA to purchase care from non-VHA providers for Veterans who live more than 40 miles from the nearest VHA facility. To explore potential impacts of these reforms on Veterans and healthcare providers, we identified VHA-users who were eligible for purchased care based on distance to VHA facilities, and quantified the availability of various types of non-VHA healthcare providers in counties where these Veterans lived.

**Methods:**

We combined 2013 administrative data on VHA-users with county-level data on rurality, non-VHA provider availability, population, household income, and population health status.

**Results:**

Most (77.9%) of the 416,338 VHA-users who were eligible for purchased care based on distance lived in rural counties. Approximately 16% of these Veterans lived in primary care shortage areas, while the majority (70.2%) lived in mental health care shortage areas. Most lived in counties that lacked specialized health care providers (e.g. cardiologists, pulmonologists, and neurologists). Counterintuitively, VHA played a greater role in delivering healthcare for the overall adult population in counties that were farther from VHA facilities (30.7 VHA-users / 1000 adults in counties over 40 miles from VHA facilities, vs. 22.4 VHA-users / 1000 adults in counties within 20 miles of VHA facilities, *p* < 0.01).

**Conclusions:**

Initiatives to purchase care for Veterans living more than 40 miles from VHA facilities may not significantly improve their access to care, as these areas are underserved by non-VHA providers. Non-VHA providers in the predominantly rural areas more than 40 miles from VHA facilities may be asked to assume care for relatively large numbers of Veterans, because VHA has recently cared for a greater proportion of the population in these areas, and these Veterans are now eligible for purchased care.

## Background

Veterans Health Administration (VHA) is the largest integrated healthcare delivery system in the United States (US), with ~ 5.9 million Veterans using VHA for some form of healthcare in 2015 [1]. Veterans are more likely than the overall United States (US) population to live in rural areas. Depending on the method used to define rural areas, 22–30% of VHA enrollees live in rural areas, compared to 15–20% of the US population [2, 3]. This makes VHA an important provider of healthcare in rural areas of the US.

VHA has worked to improve access to care for rural Veterans using a variety of strategies, including building of Community Based Outpatient Clinics (CBOCs) in rural communities, reimbursing Veterans for travel to VHA care sites, and promoting use of telehealth [4]. A more recent strategy for improving access relies on purchasing of care from non-VHA providers in communities closer to Veterans’ homes. Following a series of highly-publicized events surrounding problems with access to care, Congress enacted the Veterans Access, Choice, and Accountability Act of 2014 (aka the “Choice Act”) [5]. This act directed VHA to offer to purchase care from non-VHA providers for Veterans who live more than a 40-mile drive from the nearest VHA care site, or who are unable to obtain needed care in VHA within a “reasonable period” (i.e. generally within 30 days). Eligibility for purchased care by the 40-mile criterion is particularly relevant to Veterans living in rural areas of the US.

The transformation of VHA from a deliverer of care to a purchaser of care will have implications for both rural Veterans and the broader rural healthcare delivery system in the US. From the point of view of Veterans and VHA, a challenge will be to identify and contract with non-VHA providers in communities where Veterans live. This may be particularly difficult in communities that are already underserved by healthcare providers. From the point of view of the overall health care delivery system and non-VHA providers in rural areas, the challenge will be to accommodate an influx of patients previously cared for by VHA, which has disproportionately served rural areas.

To better understand potential challenges associated with purchasing of care for rural Veterans under the Choice Act, we identified VHA-users who were eligible for purchased care based on distance to VHA facilities, and quantified the availability of various types of non-VHA providers in counties where these Veterans lived. We also characterized areas where Veterans were eligible for purchased care, including measures of rurality, household income as a proxy for socioeconomic status, population health status, and the density of VHA-users in the overall population.

## Methods

### Data sources

We gathered person-level data on VHA-users from national VHA enrollment files for the year 2013, and linked it to county-level data on: 1) non-VHA provider availability from the Area Health Resource File (AHRF) maintained by the Health Resources and Service Administration (HRSA); 2) median household income and total adult population over age 18 from the American Community Survey (ACS) fielded by the US Census Bureau; 3) rurality based on Urban Influence Codes (UIC) created by the Economic Research Service of the US Department of Agriculture; and 4) health status measures from the Robert Wood Johnson County Health Rankings [6–8]. We compiled data for 3107 counties in the contiguous US. VHA-users and counties in Alaska and Hawaii were excluded because of the relatively small numbers of Veterans and unique geography in these states.

A VHA-user was defined as any Veteran who accessed any VHA care in 2013 in inpatient or outpatient medical, mental health, or substance use treatment settings. We determined whether individual VHA-users were eligible for non-VHA care under the Choice Act, based on their estimated driving distance to the nearest VHA care site that provided any form of inpatient or outpatient care (i.e. < 40 miles or >  40 miles). We used Federal Information Processing Standards (FIPS) county codes to link each VHA user to data on county of residence.

Data on non-VHA health care providers were obtained from the 2013 Area Health Resource File, a publicly-available data set provided by the US Health Resources and Services Administration (HRSA) [6]. Counties were classified as health professional shortage designations for primary care and mental health care using criteria created by HRSA, based on provider-to-population ratios [9]. Primary care provider (PCP) shortage areas were defined as < 1 PCP per 3500 persons. Mental health care shortage areas were defined as either: 1) < 1 psychiatrist / 20,000 persons and < 1 PCP / 6000 persons; OR 2) < 1 PCP / 9000 persons; OR 3) < 1 psychiatrist / 30,000 persons.

We also determined the availability of various non-VHA providers of specialized care in each county, including psychiatrists, cardiologists, pulmonologists, neurologists, and physical medicine and rehabilitation (PM&R) specialists. Only non-federal physicians involved in patient care were counted. We also determined the number of community health centers and community mental health centers for each county. Community health centers were limited to HRSA-grantees and community mental health centers were limited to certified Medicare providers. All physician and facility measurements were from 2013. Availability of specialty providers and facilities in each county were categorized as any or none.

County-level rurality was classified using Urban Influence Codes (UIC) created by the US Department of Agriculture’s Economic Research Service [8]. Following a commonly-applied framework, we collapsed the 12 codes into a four level measure of rurality: 1) metropolitan / (i.e. counties with population clusters > 50,000 people, UIC 1–2); 2) non-metropolitan / –adjacent to metropolitan areas (UIC 3–7); 3) nonmetropolitan - micropolitan (i.e. not adjacent to metropolitan counties but with town/urban cluster of 10,000–50,000 people, UIC 8); and 4) nonmetropolitan - remote (i.e. the remainder, UIC 9–12). For simplicity, we refer to these groups as metropolitan, rural – adjacent, rural-micropolitan, and rural-remote [8].

We used median household income as a county-level proxy for socioeconomic status. Median household income values were single-year, model-based estimates from the 2009–2013 Small Area Income and Poverty Estimates (SAIPE) provided by the US Census Bureau [10]. Based on inspection of county-level distributions of median yearly household income, we categorized counties as median household income < $30,000 (i.e. roughly bottom decile of counties), $30–$40,000; $40,000–$50,000; $50,000–$60,000; and > $60,000 (i.e. top decile of counties).

County-level health status measures were drawn from the County Health Rankings & Roadmaps program, a collaboration between the Robert Wood Johnson Foundation and the University of Wisconsin Population Health Institute [7]. As a proxy for population health status, we used age-adjusted years of potential life lost (YPLL) per 100,000 people, aggregated over three years (2010–2012). The reference age was 75. For example, a person who dies at age 45 would contribute 75–45 = 30 YPLL. The county-level YPLL is a sum of individual YPLL over all premature deaths in a county. Rates per 100,000 people were given after adjusting for differences in the age distribution over counties. YPLL values were missing for 134 counties, which were excluded from analyses of health status. County-level health status was categorized as Very Poor, Poor, Good, and Very Good according to quartiles of YPLL.

### Analysis

We began by examining the distributions of the overall US adult and VHA-user populations according to characteristics of counties of residence, including non-VHA provider availability, rurality, household income, and health status. We further stratified the VHA-user population according to eligibility for purchased care under the Choice Act based on driving distance to the nearest VHA care site (i.e. <  40 miles or >  40 miles). We repeated analyses for the subset of all VHA-users living in rural counties. We used Chi-square tests to compare distributions across county categories.

Because non-VHA providers will be more impacted by reforms to purchase care for Veterans in areas where VHA has recently delivered care for larger portions of the overall population, we also calculated the density of VHA-users in the total adult population (i.e. VHA-users / 1000 adults), according to county rurality and distance to the nearest VHA care site. Counties were categorized according to their distance to the nearest VHA care site by estimating the driving distance from the population-weighted centroid for each county to the nearest VHA care site. Population-weighted centroid coordinates were determined based on 2013 Census data, using the MABLE/Geocorr tool from the Missouri Census Data Center [11]. Coordinates of VHA facilities were collected from the Department of Veterans Affairs National Center for Veterans Analysis & Statistics, furnished by ESRI (http://www.va.gov/vetdata/maps.asp). Driving distances were estimated using ArcOnline [12]. Other analyses were completed using SAS software v9.2 (Cary, NC). All analyses were approved by the Institutional Review Board at the University of Iowa.

## Results

Overall, 416,338 (7.6%) of 5,511,483 VHA-users were eligible for non-VHA care under the Choice Act, based on residence more than 40-miles from the nearest VHA care site (Table 1). VHA-users who were eligible for purchased care based on distance were much more likely than the overall US population to live in counties that were any category of rural (87.9% vs. 17.1%), rural-remote (20.5% vs. 2.2%), median household income < $40,000 per year (40.4% vs. 10.7%), very poor population health status (28.4% vs. 10.3%), primary care shortage areas (15.8% vs. 4.2%), and mental health care shortage areas (70.2% vs. 22.0%). VHA-users eligible for purchased care based on distance were much less likely than the general population to live in counties with median household income over $60,000 per year (4.4% vs. 26.3%). All differences were statistically significant with *p* < 0.01.

**Table 1:** Percentages of overall US adult population and VHA users, by eligibility for purchased care based on distance to VHA facilities, and characteristics of county of residence

County Characteristic	% US Adult Population	% VHA users		
		Overall *N* = 5,511,483	< 40 Miles N = 5,095,145	> 40 Miles *N* = 416,338
Rurality				
Rural - Remote	2.2	3.5	2.1	20.5
Rural - Micropolitan	2.8	3.9	3.2	12.2
Rural - Metro Adjacent	10.0	13.8	11.2	45.1
Metropolitan	85.0	78.8	83.5	22.1
Median Household Income				
$0–29,999	0.4	0.6	0.3	3.6
$30,000–39,999	10.2	14.0	12.1	36.9
$40,000–49,999	31.6	38.9	38.9	39.4
$50,000–59,999	31.5	28.6	29.6	15.9
$ > 60,000	26.3	17.9	19.1	4.2
Health Status^a^				
Very Poor	8.4	11.5	10.2	28.4
Poor	15.9	21.4	21.0	26.1
Good	29.0	31.7	32.3	24.5
Very Good	46.6	35.4	36.5	21.0
Health Professional Availability				
Primary Care Shortage Area	4.0	5.0	4.2	15.8
Mental Health Care Shortage Area	20.2	25.2	21.6	70.2
County without:				
Psychiatrist	10.2	13.6	10.1	56.3
Cardiologist	12.2	16.7	12.7	65.7
Pulmonologist	15.7	21.2	16.7	76.5
Neurologist	14.6	19.4	15.2	71.1
PM&R^b^ Specialist	18.1	24.6	20.1	78.8
Community Health Center	15.1	18.2	15.8	47.0
Community Mental Health Center	57.0	62.3	59.8	93.0

^a^Age-adjusted years of potential life lost per 100,000 persons

^b^PM&R: Physical Medicine and Rehabilitation

In general, there was limited availability of non-VHA health care specialists in areas where Veterans were eligible for non-VHA care based on distance to the nearest VHA care site. The majority of VHA-users eligible for purchased care based on the 40 mile criterion lived in counties with no psychiatrists, cardiologists, pulmonologists, neurologists, PM&R specialists, or community mental health centers (Table 1). Nearly half (47%) lived in counties with no community health center.

When limiting analyses to the ~ 1.1 million VHA-users residing in rural counties, we found that 324,162 (27.8%) were eligible for purchased care from non-VHA providers based on distance to VHA care sites (Table 2). In general, availability of non-VHA providers was even more limited for rural Veterans eligible for purchased care under the Choice Act, compared to the entire population of Veterans eligible for purchased care. Availability of non-VHA mental health providers was especially limited for these rural Veterans. For example, 75.4% of rural Veterans eligible for purchased care under the Choice Act lived in counties that were mental health care shortage areas, and 73.3% in counties without psychiatrists.

**Table 2:** Percentages of rural VHA-users, by eligibility for purchased care based on distance to VHA care sites, and characteristics of county of residence

County Characteristic	% Rural VHA users		
	Overall *N* = 1,165,646	< 40 Miles *N* = 841,484	> 40 miles *N* = 324,162
Rurality			
Rural - Remote	16.4	12.5	26.4
Rural - Micropolitan	18.6	19.7	15.6
Rural - Metro Adjacent	65.0	67.8	58.0
Median household income			
$0–29,999	2.6	1.9	4.3
$30,000–39,999	37.8	36.0	42.7
$40,000–49,999	45.0	47.7	38.1
$50,000–59,999	12.8	12.8	12.7
> $60,000	1.8	1.6	2.2
Health Status			
Very poor	29.0	27.7	32.4
Poor	27.4	28.0	25.9
Good	25.9	27.2	22.4
Very good	17.7	17.1	19.3
Health Professional Availability			
Primary Care Shortage Area	12.5	11.2	15.9
Mental Health Care Shortage Area	65.5	61.7	75.4
County without:			
Psychiatrist	47.4	41.4	63.0
Cardiologist	56.5	50	73.3
Pulmonologist	69.8	63.5	85.9
Neurologist	63.6	57.4	79.7
PM&R Specialist	73.9	68.7	87.3
Community Health Center	46.5	44.3	52.2
Community Mental Health Center	92.2	91.4	94.0

**Fig. 1:**
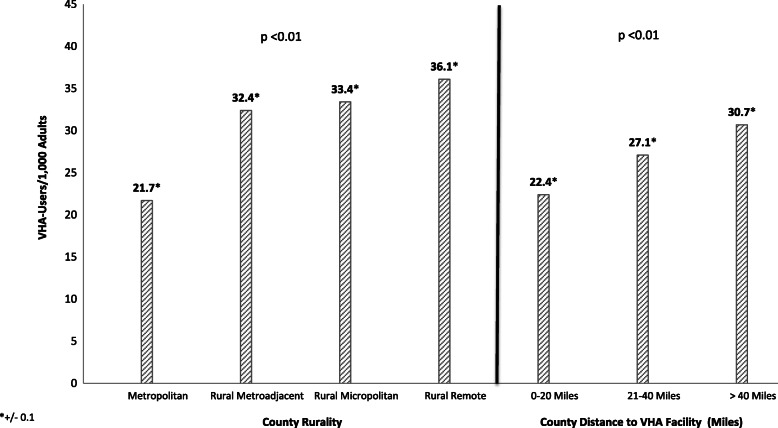
Density of VHA-Users in the total adult population over age 18 (VHA-users / 1,000 adults), by county distance to VHA facilities and rurality

To estimate the potential impact of Choice Act reforms on non-VHA providers in rural communities and areas far from VHA facilities, we next examined the density of VHA-users in the overall adult population, according to county rurality and distance from VHA care facilities. Somewhat counterintuitively, we found that VHA played a greater role in delivering care for the overall adult population in counties that were more rural and farther from VHA care sites (Fig. 1). The proportion of US adults using VHA care was overall 37% greater in counties that were more than 40 miles from VHA care sites, compared to counties within 20 miles of the nearest VHA care facility (30.7 VHA-users / 1000 adults in counties over 40 miles from VHA care vs. 22.4 / 1000 in counties within 20 miles of VHA care, *P* < 0.01). The density of VHA-users increased from 21.7 VHA-users per 1000 adults in metropolitan counties to 36.1 VHA-users per 1000 adults in rural remote counties (*p* < 0.01).

## Discussion

We found that the majority of VHA-users who were eligible for Choice Act purchased care based on distance to VHA facilities lived in rural counties. These counties were underserved by non-VHA providers, and in particular by providers of mental health and medical specialty care. Efforts to improve access to care for these Veterans by purchasing care from non-VHA providers may have limited impact, because there are relatively few non-VHA providers in these areas to provide this care.

This finding has implications for VHA efforts to improve access to care for rural Veterans. In addition to reforms to purchase non-VHA care, VHA should continue to develop other strategies for improving access to care for Veterans in areas far from VHA care sites. Examples include programs for in-home telehealth visits and subsidized transportation, both of which currently exist but could be expanded [4, 13]. VHA should particularly work to develop programs to improve access to VHA mental health and medical specialty care in rural areas, because there are few non-VHA providers in these areas to deliver this care through purchasing agreements. More generally, VHA should support broader policy efforts to increase the overall supply of health care providers in rural areas, as Veterans disproportionately live in these medically-underserved areas.

Somewhat counterintuitively, we also found that the relative role of VHA as a health care provider in the overall community – as measured by the proportion of adults using VHA care – was greater in counties that were rural and farther from VHA care sites. This was likely due to two factors. First, areas far from VHA facilities are generally rural, and residents of rural areas are more likely to join the all-volunteer military and to be Veterans [14]. Second, although Veterans in these areas must travel to obtain care in VHA care sites, there are few local, non-VHA options for care. Healthcare providers in the predominantly rural areas that are more than 40 miles from VHA care sites will likely be asked to assume care for relatively large numbers of Veterans currently using VHA care. This is true both because VHA has recently cared for a larger proportion of the population in these areas compared to counties closer to VHA facilities, and because Veterans in these areas are now generally eligible for purchased care under the Choice Act. However, we found that there are few non-VHA providers in these areas to take on care for Veterans. Taken together, these findings indicate that VHA reforms to purchase care may stress already overburdened rural providers.

The majority of VHA-users eligible for purchased care based on distance lived in counties that were not only rural and underserved by non-VHA providers, but also lower income and lower health status. Reforms that move VHA towards purchasing care should include efforts to strengthen existing safety net providers in these low income and low health status areas, so that they are better able to care for Veterans currently using VHA care. Others have previously noted the role of VHA as a safety net provider in our national health care system, and this must be kept in mind during reforms that move VHA away from delivering care and towards purchasing of care [15].

Our analyses have limitations. First, there is potential for ecological fallacy in county-level analyses. Associations apparent at the county-level may not hold at the individual level. This is an inherent challenge in using county-level data, which was necessary to combine the mostly county-level data sources used in our analyses. For the same reason, we used a county-based measure of rurality using urban influence codes. A rurality measure using a smaller area unit may provide somewhat different results. In addition, we examined availability of non-VHA providers at the county-level, and in some cases providers may have existed in relatively nearby communities in neighboring counties. The administrative data on VA enrollees were from 2013, which were the most recent data available at time analyses were initiated. The geographic distribution of Veterans is subject to change over time.

Future studies of initiatives to improve access for rural Veterans by purchasing care from community providers should evaluate the balance between community provider availability and care needs of Veterans in smaller, defined rural regions in the US. In addition, these studies should evaluate the impact of purchased care on overall healthcare use and outcomes for rural Veterans.

## Conclusions

The majority of VHA-users eligible for purchased care based on distance to VHA facilities lived in counties that were rural, underserved by non-VHA providers, lower income, and lower health status. It may often be difficult for VHA to purchase care for Veterans living more than 40 miles from VHA facilities, because these areas are already underserved by non-VHA providers. VHA should continue to develop telehealth programs to deliver care to Veterans in rural areas underserved by both community and VHA providers. Such programs are a necessary complement to initiatives to purchase in-person care from community providers.

## Abbreviations

ACS: American Community Survey
AHRF: Area Health Resource File
CBOC: Community Based Outpatient Clinic
HRSA: Health Resources & Services Administration
PCP: Primary Care Provider
PM&R: Physical Medicine & Rehabilitation
UIC: Urban Influence Code
US: United States
VHA: Veterans Health Administration
YPLL: Years of Potential Life Lost
