# Correction to: “Availability of healthcare providers for rural veterans eligible for purchased care under the veterans choice act”

**DOI:** 10.1186/s12913-020-05983-z

**Published:** 2020-12-22

**Authors:** Michael E. Ohl, Margaret Carrell, Andrew Thurman, Mark Vander Weg, Teresa Hudson, Michelle Mengeling, Mary Vaughan-Sarrazin

**Affiliations:** 1 VA Office of Rural Health (ORH), Veterans Rural Health Resource Center Iowa City, Iowa City VA Medical Center, Iowa City, IA USA; 2 Center for Comprehensive Access and Delivery Research and Evaluation (CADRE), Mailstop 152, Iowa City VAMC, 52246m, Iowa City, IA USA; 3 Department of Internal Medicine, University of Iowa Carver College of Medicine, Iowa City, IA USA; 4 Department of Geographical and Sustainability Sciences, University of Iowa, Iowa City, IA USA; 5 Center for Mental Healthcare and Outcomes Research, Central Arkansas VA, Little Rock, AR USA

**Correction to: BMC Health Serv Res 18, 315 (2018)**

**https://doi.org/10.1186/s12913-018-3108-8**

Following publication of the original article [1], the authors identified an error in the author name of Teresa Hudson.

The incorrect author name is: Teresa Hudson Pharm

The correct author name is: Teresa Hudson

The author group has been updated above, and the original article has been corrected.
